# Validation of AIST‐SHANEL Model Based on Spatiotemporally Extensive Monitoring Data of Linear Alkylbenzene Sulfonate in Japan: Toward a Better Strategy on Deriving Predicted Environmental Concentrations

**DOI:** 10.1002/ieam.4167

**Published:** 2019-08-09

**Authors:** Tohru Nishioka, Yuichi Iwasaki, Yuriko Ishikawa, Masayuki Yamane, Osamu Morita, Hiroshi Honda

**Affiliations:** ^1^ R&D Safety Science Research, Kao Corporation Tochigi Japan; ^2^ Research Institute of Science for Safety and Sustainability National Institute of Advanced Industrial Science and Technology Ibaraki Japan

**Keywords:** AIST‐SHANEL, Exposure assessment, LAS, River, Simulation model, Surfactant

## Abstract

Strategies for deriving predicted environmental concentrations (PECs) using environmental exposure models have become increasingly important in the environmental risk assessment of chemical substances. However, many strategies are not fully developed owing to uncertainties in the derivation of PECs across spatially extensive areas. Here, we used 3‐year environmental monitoring data (river: 11 702 points; lake: 1867 points; sea: 12 points) on linear alkylbenzene sulfonate (LAS) in Japan to evaluate the ability of the National Institute of Advanced Industrial Science and Technology (AIST)‐Standardized Hydrology‐Based Assessment Tool for the Chemical Exposure Load (SHANEL) model developed to predict chemical concentrations in major Japanese rivers. The results indicate that the estimation ability of the AIST‐SHANEL model conforms more closely to the actual measured values in rivers than it does for lakes and seas (correlation coefficient: 0.46; proportion within the 10× factor range: 82%). In addition, the 95th percentile, 90th percentile, 50th percentile, and mean values of the distributions of the measured values (14 µg/L, 8.2 µg/L, 0.88 µg/L, and 3.4 µg/L, respectively) and estimated values (19 µg/L, 13 µg/L, 1.4 µg/L, and 4.2 µg/L, respectively) showed high concordance. The results suggest that AIST‐SHANEL may be useful in estimating summary statistics (e.g., 95th and 90th percentiles) of chemical concentrations in major rivers throughout Japan. Given its practical use and high accuracy, these environmental risk assessments are suitable for a wide range of regions and can be conducted using representative estimated values, such as the 95th percentile. *Integr Environ Assess Manag* 2019;15:750–759. © 2019 The Authors. Integrated Environmental Assessment and Management published by Wiley Periodicals, Inc. on behalf of Society of Environmental Toxicology & Chemistry (SETAC).

## Key Points

- The performance of an environmental exposure model, AIST–SHANEL, for estimating chemical concentration in rivers was evaluated using comprehensive measurement data from major rivers throughout Japan.
- The estimated spatiotemporal LAS concentrations, especially the 95th and 90th percentiles, were in good agreement with the measured concentrations.
- Using representative estimated values, e.g., 95th percentile, AIST–SHANEL can precisely estimate chemical concentrations in major rivers throughout Japan, and can contribute to high–accuracy environmental risk assessments.

## INTRODUCTION

Since the adoption of the Strategic Approach to International Chemicals Management (SAICM) framework at the International Conference on Chemicals Management in 2006, risk assessments for the management of chemicals have been actively conducted worldwide (Hase and Kitano 2012). Environmental risk assessments for various chemicals are ongoing because understanding exposure situations in rivers across a wide range of regional conditions is important. In general, chemicals that are routinely measured by environmental monitoring are limited; therefore, actual environmental concentrations are unavailable for most chemicals. In addition, even for chemicals that have been monitored, the study duration and study area are often limited. Thus, a highly or at least reasonably accurate exposure assessment model is needed to estimate spatiotemporal exposure levels for a wide range of regions.

Exposure assessment models for chemicals are being developed worldwide. In Europe and North America, different models have been developed based on the Uniform Substance Evaluation System (USES), including the European Union System for the Evaluation of Substances (EUSES; Vermeire et al. 1997), a level III fugacity (i.e., non‐equilibrium, steady state with advection) dynamic predictive simulation model, and the Risk Assessment, Identification and Ranking (RAIDAR) model (Wambaugh et al. 2013), a Canadian level II fugacity (i.e., equilibrium, steady state with advection) multimedia model. Furthermore, the Geography Referenced Regional Environmental Exposure Assessment Tool for European Rivers (GREAT‐ER; Cefic‐LRI 2017) and the Better Assessment Science Integrating Point and Nonpoint Sources (BASINS; United States Environmental Protection Agency 2015) have been published for environmental risk assessment and management of chemicals using Geographic Information System (GIS) applications. In addition, georeferenced multimedia environmental fate models with detailed spatial resolutions have been developed by the National Institute for Environmental Studies (NIES) in Japan, including the Grid‐Catchment Integrated Environmental Modeling System (G‐CIEMS; National Institute for Environmental Studies 2014) and the National Institute of Advanced Industrial Science and Technology (AIST)‐Standardized Hydrology‐Based Assessment Tool for Chemical Exposure Load (SHANEL) (AIST‐SHANEL; National Institute of Advanced Industrial Science and Technology 2015). In particular, AIST‐SHANEL can estimate the spatiotemporal distribution of chemical concentrations in river water at fine spatial scales.

Although many exposure assessment models have been developed, they have not been fully investigated in terms of accuracy using environmental monitoring data for multiple chemicals from a wide range of areas. Most exposure assessment models (Suzuki et al. 2004; Ishikawa and Tokai 2006; Kehrein et al. 2015; Kapo et al. 2016) have compared only hundreds of data points for a particular chemical. However, there are no adequate comparative analyses of the values estimated from these exposure assessment models with measured values, which may prevent their practical application.

The effectiveness of AIST‐SHANEL for exposure assessments has been evaluated by several studies. For instance, we have reported on 5 surfactants (linear alkylbenzene sulfonate [LAS], amine oxide [AO], alcohol ethoxylate, ester‐amide type dialkyl amine salt, and triethanolamine quaternary salt), and found that AIST‐SHANEL exposure assessments were effective for various chemicals (Nishioka et al. 2018). Other groups have also investigated the effectiveness of AIST‐SHANEL using surfactants such as LAS and AO, and the Japan Soap and Detergent Association has applied the model to ecological risk assessments (Yamamoto et al. 2010; Japan Soap and Detergent Association 2010, 2011; Ishikawa et al. 2017). However, these studies used measured values from environmental monitoring studies at limited sites. Acquiring high‐quality environmental monitoring data from across wide areas is challenging.

LAS is a chemical compound regulated by environmental water quality standards to protect aquatic life in Japan. A nationwide environmental monitoring project has been conducted for LAS across Japan since 2013, and a large measurement dataset is now available (Ministry of the Environment, Japan 2018a). Therefore, the aim of the present study was to evaluate the estimation accuracy of AIST‐SHANEL as an exposure assessment model for major rivers throughout Japan. We compared the AIST‐SHANEL‐estimated concentrations of LAS with actual measured concentrations in terms of individual data, annual site mean data, their quantile points, monitoring study site attributes (river, lake or marsh, and sea), and the model characteristics. Although many input parameters have been used in the model, traction coefficient (bed‐load transport coefficient), a unique parameter representing wet‐weather effluent load in the sediment, is known as a major contributing factor (Nishioka et al. 2018). Therefore, influence of the traction coefficient on concordance was also evaluated. Finally, using the results, we attempted to develop a strategy for the effective application of AIST‐SHANEL to environmental risk assessments.

## METHODS

### Measured environmental LAS concentrations

We used 23 305 data points of LAS monitoring in public waters from rivers (12 217 data points), lakes (2414 data points), and the sea (3674 data points) in Japan during 3 years (2013–2015). The data were obtained from the Ministry of the Environment, Japan (Ministry of the Environment, Japan 2018a). The range of reported LAS concentrations was 0.06 to 800 µg/L. Regarding location information on water collection sites in the monitoring studies, the absolute serial number set by the NIES was converted into latitude and longitude information, which was then converted into a tertiary mesh code. Using the tertiary mesh code, we were able to compare monthly river water concentrations, estimated using AIST‐SHANEL at each location. In cases when multiple monitoring studies were conducted at the same site, the mean values for each year were calculated. Sampling sites were different depending on the year because the total number of sampling points was not the same for each year. However, in general, almost all points evaluated in the previous year were evaluated the following year. Moreover, the sampling frequency at individual points of the rivers is as follows: mean: 6.2 times per year; median: 4 times per year; maximum: 72 times per year; minimum: once per year (see Supplemental Data for more details).

### Environmental exposure assessment model

The LAS concentrations in river water were calculated using the tertiary mesh class A river system edition in AIST‐SHANEL (version 3.0), which was released by AIST in 2015 (Ishikawa et al. 2017). Class A rivers are major rivers defined by the River Law (Ministry of Land, Infrastructure, Transport and Tourism, Japan 2006) and managed by the Ministry of Land, Infrastructure and Transport, Japan. Currently, 109 rivers are classified Class A. In the present study, a Class A river is presented as a major river unless otherwise noted. AIST‐SHANEL can estimate the chemical exposure concentrations in a water system with a grid spatial resolution of 1 × 1 km and a monthly temporal resolution based on river basin information, point or nonpoint emission data, and basic physicochemical properties of the chemical substances of interest. A variety of information can be easily derived using this tool, including the location of high‐emission areas within river basins, the correspondence between exposure loads and areas with high emissions, and the relationship between river flow rate and load.

AIST‐SHANEL estimates the chemical discharge that does not pass through sewage treatment plants (i.e., site discharge) in virtual watershed channels, specifically the inflow of gray water from houses, excluding pesticides. In addition, it models the runoff and deposition and traction processes due to biodegradation via distribution equilibrium and microbial degradation of dissolved and suspended matter, as well as anthropogenic wastewater drainage. The process of deposition and traction is considered the movement of matter that has accumulated in watershed channels by traction when water levels rise. The runoff load in rivers due to the deposition and traction process is indicated by Equations 1 and 2 (Ishikawa and Tokai 2006; Ishikawa et al. 2012; Ishikawa et al. 2017; Nishioka et al. 2018):

(1) $$fr_{\text{n}\text{p}}=kw_{\text{n}\text{p}}S_{\text{n}\text{p}}\frac{q_{\text{a}\text{l}}}{A}$$

(2) $$\frac{dS_{\text{n}\text{p}}}{dt}=\left(1-f_{\text{p}}\right)x_{\text{m}\text{p}}-fr_{\text{n}\text{p}}-K_{\text{n}\text{p}}S_{\text{n}\text{p}}$$

where *f*_*p*_ is the mass distribution ratio of human wastewater in water channels and bottom sediments (unitless), *x*_mp_ is the site discharge (mg s^−1^), *fr*_np_ is the runoff load (mg s^−1^), *S*_np_ is the sediment load (mg), *kw*_np_ is the traction coefficient (m^−1^), *q*_al_ is the flow rate (m^3^ s^−1^), *A* is the watershed area (m^2^), and *K*_np_ is the degradation speed (s^−1^).

Because the traction coefficient has been reported to influence estimation accuracy, it was calibrated using 5 surfactants, including LAS (Nishioka et al. 2018). To identify the best conditions for the traction coefficient for assessment across a wide area, we used values of 0.01, 30, 100, 300, and 1000 m^−1^, as assessed by Nishioka et al. (2018).

### Estimated environmental LAS concentrations

As in previous reports (Ishikawa et al. 2017; Nishioka et al. 2018), the monthly estimated LAS concentrations were calculated for major rivers throughout Japan (on a 1‐km grid) using AIST‐SHANEL version 3.0. LAS is a chemical mixture in which the number of alkyl carbons in the compound varies. In this study, the number of alkyl carbons was taken to be the mean number of alkyl carbons (11.6). The analyses were conducted using previously reported physical properties (Table 1) (Ishikawa et al. 2012; HERA 2013). The total of the numerical values from public data from the Pollutant Release and Transfer Register of the Ministry of the Environment, Japan in 2011 (Ministry of the Environment, Japan 2018b) was used for the total environmental discharge of LAS (Table 2). LAS is used in many household products; therefore, discharge from households comprises the majority of LAS discharge. Because sewer systems are widespread throughout Japan, about 76% of the total LAS passes through sewers. In this study, the discharge volume at every reported site was used to determine the quantity of LAS derived from point sources. The estimated unreported quantity of LAS was assigned as the proportional distribution of the estimated discharge volume by region based on population distribution, and this value was used as nonpoint source information.

**Table 1:** Physicochemical properties of LAS used in AIST‐SHANEL model.

Parameter	Value
Vapor pressure (Pa)	3.05 × 10^‐13^ ^a^
Molecular weight (g/mol)	342.4^b^
Water solubility (g/m^3^)	2.5 × 10^b^
Organic carbon–water partition coefficient *K*_oc_ (L/kg)	2500^b^
Half‐life in river water (day)	0.75^b^
Half‐life in sediment (day)	22^a^
Half‐life in soil (day)	14^a^
Removal in sewage treatment plants (unitless)	0.99^a^

LAS = linear alkylbenzene sulfonate; AIST‐SHANEL = Advanced Industrial

Science and Technology (AIST)‐Standardized Hydrology‐Based Assessment

Tool for the Chemical Exposure Load (SHANEL).

^a^ Ishikawa et al. 2012.

^b^ HERA 2013.

**Table 2:** LAS emission levels used in AIST‐SHANEL model

PRTR classification	Emissions into public waters (ton/year)	Amount transferred into sewage treatment plants (ton/year)
Reported quantity	15	29
Estimated unreported quantity: Nontarget companies	67	127
Estimated unreported quantity: Nontarget industrial and household use	10 412	33 005

LAS = linear alkylbenzene sulfonate; AIST‐SHANEL = Advanced Industrial; Science and Technology (AIST)‐Standardized Hydrology‐Based Assessment; Tool for the Chemical Exposure Load (SHANEL); PRTR = pollutant release and transfer; Register.

### Estimation of model accuracy

The tertiary mesh codes of locations from the estimated concentrations of LAS obtained from AIST‐SHANEL using environmental monitoring information were selected, and the corresponding monthly measured concentrations from the environmental monitoring study were extracted for comparison. If there were multiple data points for the same site and year, the mean value for each site was calculated as the regional annual mean value. Furthermore, if estimated values calculated by the model were below the lower limit of detection (LOD) of 0.06–0.1 µg/L, they were replaced with a value of 0.01 µg/L to match the order of the minimum concentration of actual measured values.

Next, logarithmic conversion of the measured and estimated values was conducted, and we compared the measured concentrations or monthly averages with the corresponding monthly model estimates. The agreement between them was quantitatively assessed for every analysis condition. Subsequently, the analyses of spatiotemporal data and annual site mean data were conducted.

For the analyses, Spearman's rank correlation coefficient (*ρ*), root mean square logarithmic error (RMSLE), and the proportion of sites where the estimated values fell outside the 0.1 to 10‐fold (10× factor) range of the measured values were calculated for each water body (i.e., total, rivers, and lakes) and analysis condition (i.e., traction coefficient = 0.01–1000 m^‒1^). The measured and estimated values were judged to be relatively valid when Spearman's ρ value was 0.3 or higher. The RMSLE and 10× factor values were calculated to enable relative comparisons of the estimation accuracy between models, where smaller values, as a result of changes in model conditions, were indicative of higher estimation accuracies. The RMSLE was calculated as described previously (Imaizumi et al. 2018):

(3) $$\text{RMSLE}\;=\;\sqrt{\frac{{\sum \left(\log_{10}P-\log_{10}O\right)}^{2}}{n}}$$

where *P* is the predicted concentration (µg/L), *O* is the observed concentration (µg/L), and *n* is the number of pairs of predicted and observed concentrations.

For the analysis conditions with the highest estimation accuracy, scatter plots were created for the measured and estimated values classified by water body (total, rivers, and lakes) for all spatiotemporal data and annual site mean data.

### Estimation of concentration distributions in river water and determination of risk assessment strategy

When using an exposure assessment model to conduct environmental risk assessments of chemicals discharged into waters, it is necessary to derive a high concentration value that can cover the majority of public water bodies. This value is considered to be the predicted environmental concentration (PEC). Evaluations for environmental risk assessments are conducted by comparing the derived PEC and the concentration that is predicted not to have a harmful effect on the ecosystem (i.e., the predicted no‐effect concentration; European Communities 2003). Consequently, in this study, the distribution of measured values and AIST‐SHANEL‐estimated values were compared using box plots (plotting the maximum, 95th percentile, 90th percentile, 50th percentile, 10th percentile, 5th percentile, and minimum values) for each analytical dataset (all spatiotemporal data and annual site mean data) and water body (total and rivers). Then, we created a strategy to derive the PEC using AIST‐SHANEL.

## RESULTS

### Estimation ability of LAS concentrations in river water

The monthly mean LAS concentrations in river water at 112 434 sites (1‐km mesh) were estimated using AIST‐SHANEL version 3.0 (1 349 208 data points in total). In addition, the monthly environmental monitoring data of 13 581 measurements (river: 11 702; lake: 1867; sea: 12) from 2013 to 2015 were obtained. We calculated 2202 annual site mean data points for individual sites (river: 1995; lake: 203; sea: 4).

**Figure 1:**
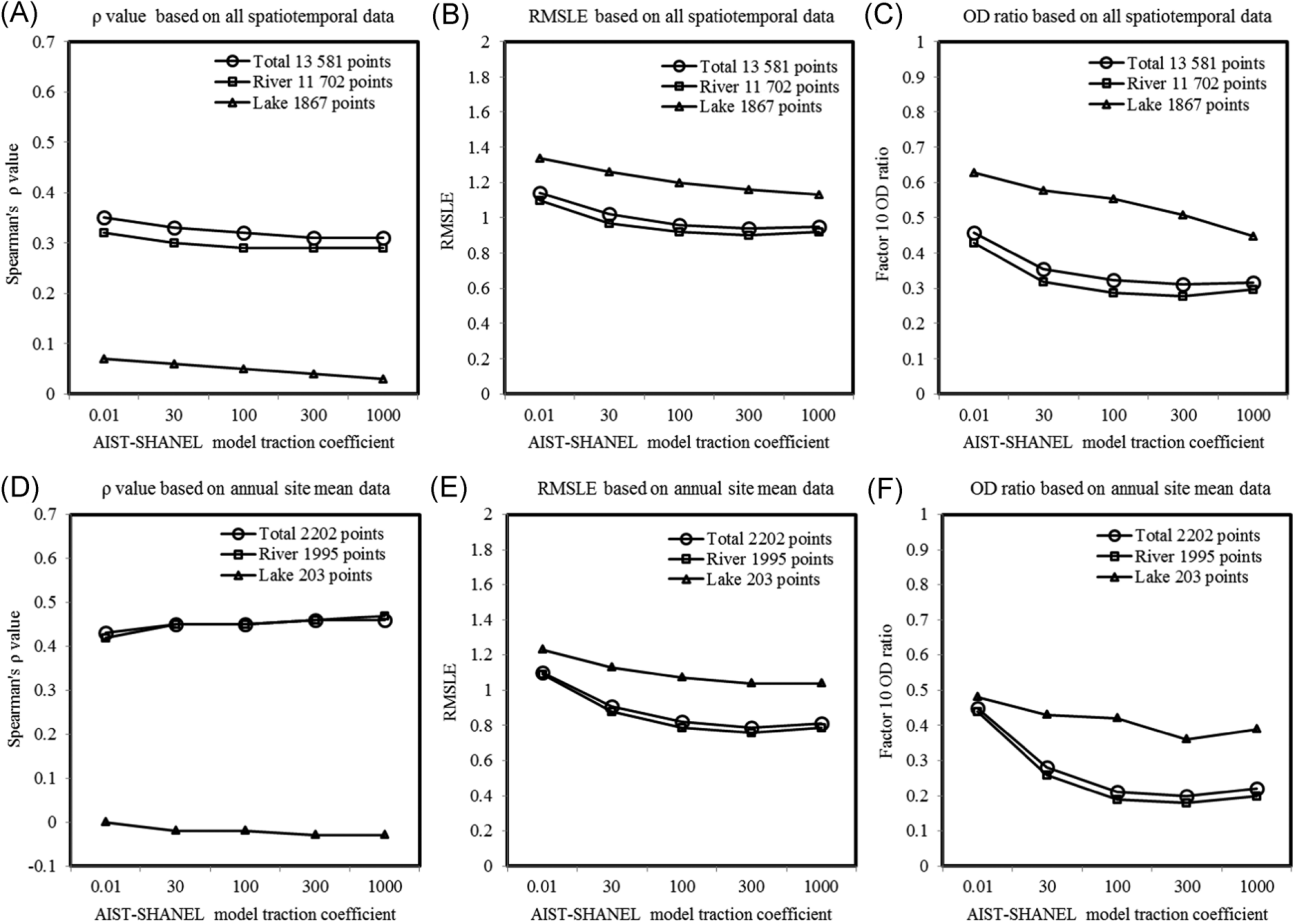
Estimation accuracy of linear alkylbenzene sulfonate (LAS) concentrations using the AIST‐SHANEL model. Evaluation using all spatiotemporal data (**A, B, C**); evaluation using annual site mean data (**D, E, F**). Evaluation using Spearman's ρ values (**A, D**); evaluation using the root mean square logarithmic error (RMSLE) (**B, E**); evaluation using the 10× factor out‐of‐domain (OD) ratio (**C, F**).

The accuracy of the model using statistical indicators for each analytical data type (all spatiotemporal and annual site mean data) and water body (total, rivers, and lakes) was quantitatively evaluated (Figure 1). No statistical analysis was conducted for sea regions due to the small sample size. First, the correlations between the measured and estimated values (i.e., Spearman's ρ values) using all spatiotemporal data showed weak correlations for the total data and river data regardless of the traction coefficient value (Figure 1A and 1D; *ρ* = 0.29–0.35). In addition, almost no correlations were observed for the lake data regardless of the traction coefficient value (ρ = 0.03–0.07). A similar analysis using the annual site mean data showed higher positive correlations for the total data and river data (ρ = 0.42–0.47) but almost no correlations for the lake data (ρ = −0.03–0.00).

Next, the error between the measured and estimated values using the RMSLE was evaluated (Figure 1B and 1E). In the assessment using all the spatiotemporal data, the RMSLE for the total data was less than 1 for traction coefficients of 100 m^‒1^ and higher, with the minimum value (0.94) being observed at 300 m^‒1^. Similarly, the RMSLE for the river data was less than 1 for traction coefficients of 30 m^‒1^, and the minimum value (0.90) was observed at 300 m^‒1^. In contrast, for the RMSLE of lake data, higher traction coefficients were associated with smaller RMSLE values, although the RMSLE ranged from 1.13 to 1.34; therefore, the lake data had the largest error. Similar results were obtained for the annual site mean data, and the error values were smaller compared with all spatiotemporal data (minimum values: total, 0.79; river, 0.76; lake, 1.04).

Finally, the number of estimated outliers based on the proportion of data outside the 10× factor range was evaluated (Figure 1C and 1F). The assessment based on all spatiotemporal data showed a proportional increase in RMSLE as the proportion of data outside the 10× factor range increased. The ranges of the values were as follows: 0.31 to 0.46 for total data (minimum value at a traction coefficient of 300 m^‒1^), 0.28 to 0.43 for river data (minimum value at a traction coefficient of 300 m^‒1^), and 0.45 to 0.63 for lake data (minimum value at a traction coefficient of 1000 m^‒1^). The results of a similar assessment using annual site mean data showed that the proportion of data outside the 10× factor range decreased for all water bodies; as a result, the concordance increased compared with grab sample data (minimum values: total, 0.20; river, 0.18; lake, 0.36).

These results revealed low estimation accuracy for lake data. Due to the small sample size of the lake data, such low accuracy had a limited influence on the estimation ability of the total data; regardless, using river data alone increased the estimation accuracy. In addition, using the annual site mean data resulted in an increase in agreement versus using all spatiotemporal data owing to the averaging of outliers. However, even for the analysis conditions with the best agreement (annual site mean data, river data, traction coefficient of 300 m^‒1^), the correlation coefficient was 0.46, and 18% of data fell outside the 10× factor range. Therefore, some issues were noted regarding the estimation of chemical concentrations with high accuracy at individual sites.

### Measured versus estimated values

**Figure 2:**
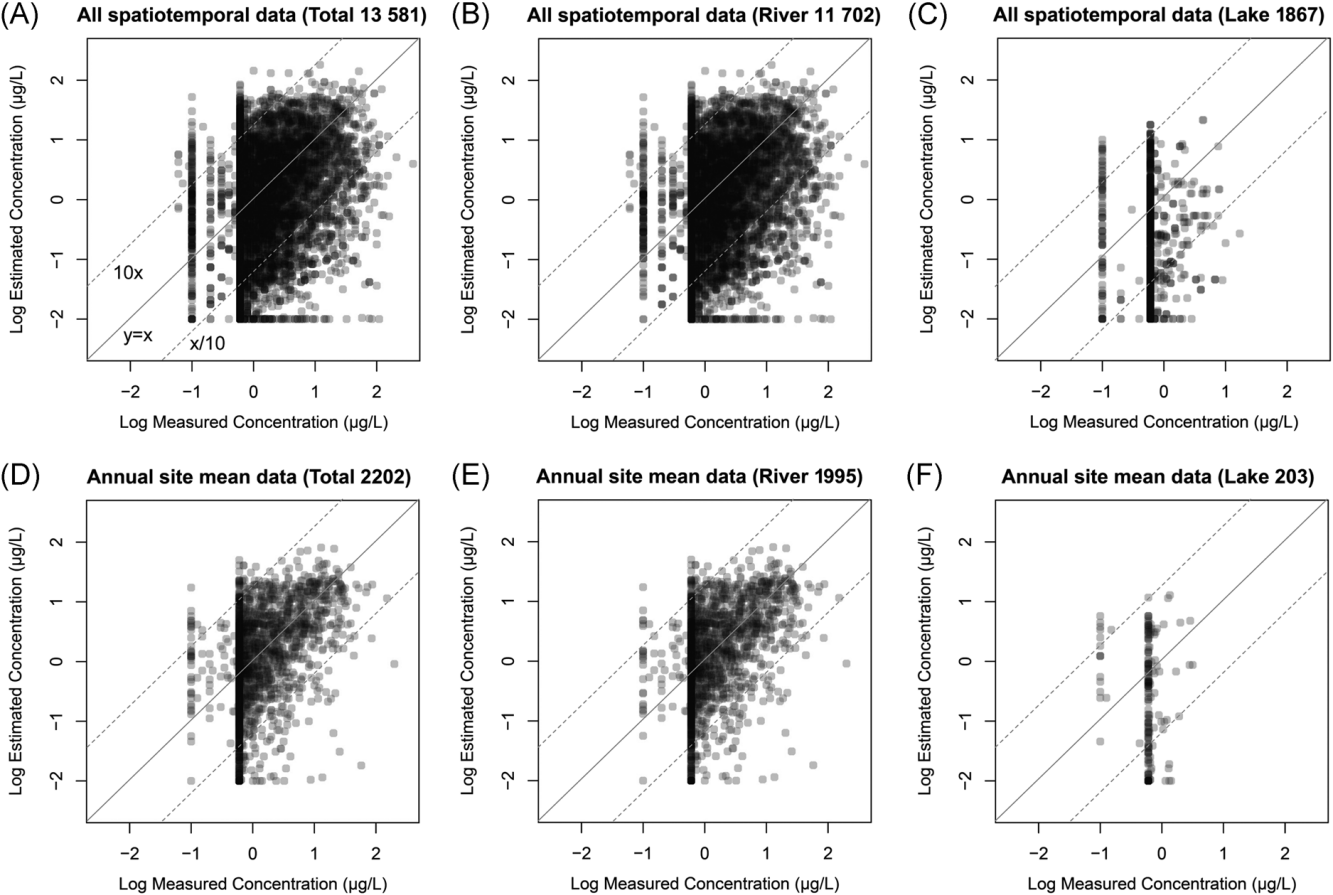
Measured linear alkylbenzene sulfonate (LAS) concentrations versus concentrations estimated with the AIST‐SHANEL model. Comparison of all spatiotemporal data, including (**A**) total (13 581 data points), (**B**) river (11 702 data points), and (**C**) lake (1867 data points) data. Comparison of annual site mean data, including (**D**) total (2202 data points), (**E**) river (1995 data points), and (**F**) lake (203 data points) data. The traction coefficient of the AIST‐SHANEL model was 300 m^‐1^. In all Figure 2 sections, the solid gray line is the 1:1 line, and the upper and lower dashed lines represent 10 and 1/10 times the 1:1 line, respectively.

Scatter plots of log‐transformed measured and estimated values for the best analysis condition identified (traction coefficient of 300 m^−1^) were used to confirm the individual spatiotemporal data (Figure 2). The plots using all spatiotemporal data classified by water body (total, river, and lake) showed relatively good agreement between the measured and estimated values for the total data and river data given that more than 80% of the estimates were within a factor of 10 of the measured values (Figure 2A and 2B). In contrast, for the lake data, the proportion of measured values plotted near the LOD (0.06–0.6 µg/L) was higher than that for the river data, making it difficult to conduct a sufficient comparison with the estimated values; therefore, the agreement was determined to be low (Figure 2C).

Similarly, for the plots using the annual site mean data classified by water body (total, river, and lake), the total, river, and lake data tended to have fewer outliers compared with the analysis of all spatiotemporal data (Figure 2D, 2E, and 2F). For example, the maximum concentration from the environmental monitoring, 800 µg/L LAS, was obtained from 1 measurement at a single site (tertiary mesh code: 53361646, January 2014), whereas the mean LAS concentration of four measurements at that site was 200.5 µg/L, which reduced the difference between the measured and estimated values (annual mean estimate: 0.91 µg/L). In addition, the second highest concentration from environmental monitoring, 390 µg/L LAS, was observed at another site (tertiary mesh code: 53407458, October 2014), but the mean of 10 measurements at that site was 85.7 µg/L, which decreased the difference from the mean estimated value of 2.47 µg/L. These examples illustrate that because environmental monitoring concentrations are measured at a specific point in time, when a value that is substantially different than the mean is recorded, the separation between the data and AIST‐SHANEL‐estimated monthly mean increases. In addition, some (~55%) of the measured values were at the LOD. Therefore, the lack of agreement between the measured and estimated values results from the imperfect estimation ability of the model as well as the lack of representative measured values. Notably, many of the sites with substantial differences between the measured and estimated concentrations were marked by multiple river convergence, located near campsites or hot spring facilities, in rivers with very low flow rates, or near dams or lakes (Table 3). However, their differences might have been related to other reasons, including inaccurate estimation of flow rates and LAS loads at individual sampling sites, although such information is unavailable. Because of the limitation posed by less detailed data at individual sites, further meta‐analytical investigations may be useful to clarify the reasons for the large differences that were detected.

**Table 3:** Sites with substantial differences between measured and estimated concentrations

Site	LAS (µg/L)		Ratio	Measurement frequency	Geography feature
Code	Measured	Estimated	E/M		
A	0.1	17.5	175	1	Hot spring
B	0.6	50.1	84	2	Hot spring
C	0.1	7.1	71	1	Junction of rivers
D	0.6	41.2	69	1	Dam
E	0.7	41.2	59	1	—
F	0.7	40.9	58	1	Junction of rivers
G	0.1	5.8	58	4	Dam
H	0.6	32.7	55	4	Junction of rivers
I	0.1	4.8	48	1	Junction of rivers
J	0.1	4.8	48	2	—
K	2.2	0.012	0.0055	12	—
L	3.5	0.016	0.0046	4	Small river
M	200.5	0.915	0.0046	4	—
N	2.4	0.010	0.0041	12	Junction of rivers
O	3.2	0.010	0.0031	4	—
P	20.4	0.063	0.0031	3	Junction of rivers
Q	25.5	0.031	0.0012	4	Small river
R	8.7	0.010	0.0012	12	Junction of rivers
S	13.4	0.012	0.0009	12	Golf course
T	57.2	0.018	0.0003	6	Campsite

LAS = linear alkylbenzene sulfonate; E/M = ratio of estimated to measured LAS concentrations.

### Distribution of LAS concentrations in river water

**Figure 3:**
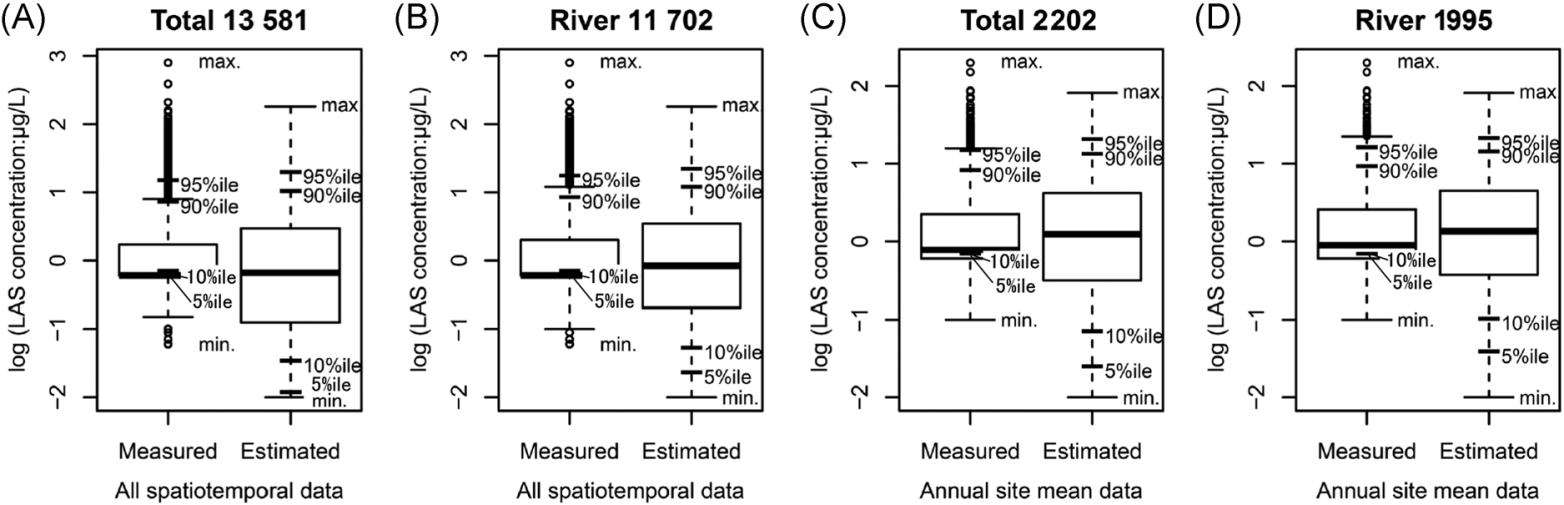
Box plots of measured linear alkylbenzene sulfonate (LAS) concentrations and the concentrations estimated with the AIST‐SHANEL model. Total (13 581 data points) (**A**) and river (11 702 data points) (**B**) samples extracted from spatiotemporal data. Total (2202 data points) (**C**) and river (1995 data points) (**D**) samples extracted from annual site mean data.

Box plots (Figure 3) were used to compare measured and estimated LAS concentration distributions for the analytical data (all spatiotemporal data and annual site mean data) and for each water body type (total and river). Because the majority of the total dataset comprised river data (86%, based on all spatiotemporal data; 91%, based on annual site mean data), no clear difference between the total data and river data was observed.

There were many outliers in the measured values for all spatiotemporal data. The maximum and minimum values tended to have low concordance with the estimated values (e.g., maximum: total measured, 800 µg/L; total estimated, 182 µg/L). In addition, the 5th and 10th percentiles of the distributions had low agreement (e.g., 5th percentile: total measured = 0.6 µg/L; total estimated = 0.01 µg/L). Although this result implies the underestimation of environmental concentrations, as shown in Figure 2, more than half of the low concentration data were near the LOD. Thus, the low agreement observed at lower percentiles was likely caused by the analytical limits of measurements rather than the inaccurate model estimation. Conversely, the interquartile range (IQR) of the measured values was within the IQR of the estimated values (e.g., IQR: total measured = 0.6–1.7 µg/L; total estimated = 0.1–3.0 µg/L). Furthermore, the 50th to 95th percentiles, which represent mid to high concentrations in the distribution, showed high agreement (e.g., 95th percentile, 90th percentile, 50th percentile: total measured = 13 µg/L, 6.1 µg/L, and 0.6 µg/L, respectively; total estimated = 17 µg/L, 9.0 µg/L, and 0.7 µg/L, respectively).

Using annual site mean data still showed low agreement with the estimated values for the maximum, minimum, 5th percentile, and 10th percentile, although agreement improved marginally (e.g., maximum: total measured = 200 µg/L; total estimated = 82 µg/L). Averaging data points near the LOD may have improved the distribution of low concentrations. Moreover, similarly for all spatiotemporal data, the IQR of the measured values was within the IQR of the estimated values (e.g., IQR: total measured = 0.6–2.3 µg/L; total estimated = 0.3–4.2 µg/L). Finally, the 50th to 95th percentiles also showed high agreement (e.g., 95th percentile, 90th percentile, 50th percentile: total measured = 13 µg/L, 7.2 µg/L, and 0.8 µg/L, respectively; total estimated = 18 µg/L, 12 µg/L, and 1.2 µg/L, respectively).

These results show that AIST‐SHANEL has high accuracy for mid to high concentrations, especially for river data. Additionally, in cases where there are multiple measurements for the same site, annual mean values contribute to decreasing the number of outliers and improving the estimation accuracy at individual points. The identified outliers might be associated with analytical error or missing peak concentrations. Therefore, resampling at sites that showed a large difference between the analytical and estimated values might help clarify the reason for the outliers and improve the accuracy of the model performance evaluation.

## DISCUSSION

We evaluated the ability of AIST‐SHANEL to estimate LAS concentrations in river water based on nationwide environmental monitoring data, and showed that the model can be useful for performing spatiotemporal exposure assessments within a wide region. The estimation ability of the model was particularly good in analyses focusing on annual mean river data. The optimal traction coefficient of LAS was similar to that in a previous report (Nishioka et al. 2018). Although conducting detailed analyses of individual sites posed some issues, 95th and 90th percentiles, which cover the majority of public bodies of water, could be estimated with high accuracy as the PEC, indicating that these values could be effective in environmental risk assessments.

No published investigations exist of environmental exposure model validations based on more than 10 000 data points of measured chemical concentrations in river water. Existing investigations of the validity of estimated concentrations in rivers in the United States, Europe, and Japan, calculated using exposure assessment models such as the in‐stream exposure model (iSTREEM), GREAT‐ER, G‐CIEMS, and AIST‐SHANEL, have been published; however, most of these studies used fewer than 500 data points (Suzuki et al. 2004; Ishikawa and Tokai 2006; Kehrein et al. 2015; Kapo et al. 2016; Nishioka et al. 2018). Thus, to our knowledge, in terms of the number of data points across an area, our research represents the most comprehensive comparison of measured and estimated values.

Although several quantitative evaluations of exposure models have been conducted, no uniform approach exists. Using a set of metrics similar to that in the present study, Imaizumi et al. (2018) compared spatiotemporal data for 25 herbicides at 7 sites versus a few to 10s of sites using Pesticide Chemicals High Resolution Estimation Method (PeCHREM) or G‐CIEMS, and reported good agreement (RMSLE: 0.57–9.03). In the present study, although we focused on only 1 chemical (LAS), AIST‐SHANEL was capable of estimating chemical concentrations over a wider range of river water bodies at a high resolution with similar accuracy to PeCHREM/G‐CIEMS, even when comparing more than 10 000 points of large‐scale environmental monitoring data (RMSLE: 0.90). Thus, we were able to verify the applicability of AIST‐SHANEL for estimating LAS concentrations in Japanese rivers that are characterized by fast flowing water and large changes in seasonal precipitation.

AIST‐SHANEL is an integrated model that considers water flow rate and load and chemical discharge. Its modeling ability depends on the accuracy of the input parameters, water flow rate and load, chemical discharge, and factors related to concentration analysis. Moreover, factors related to concentration analysis particularly depend on biodegradability, *K*_oc_, traction coefficient, and removal rate in sewage treatment plants. Among these parameters, estimation accuracy on the water flow rate and load has been evaluated (Ishikawa et al. 2012; Ishikawa et al. 2017), and information on the chemical discharge was extracted from a reliable public national database. In addition, the experimental values of all factors related to concentration analysis, except the traction coefficient, were reliable. Thus, calibration of the traction coefficient plays an important role. Nishioka et al. (2018) confirmed good estimation performance using a traction coefficient of 30 m^‒1^ for chemical substances with relatively small *K*_oc_ values (such as LAS), and traction coefficients of 300 to 1000 m^‒1^ for chemical substances with high *K*_oc_ values (ca. 1×10^5–6^). In detail, the modeling performance for LAS was good for traction coefficients ranging from 0.01 to 1000 m^‒1^, and particularly good for the value of 300 m^‒1^, whereas the residual sum of squares, which indicates model performance, was minimum. This finding was similar to that of the present study and, therefore, selecting criteria of traction coefficients on the basis of the *K*_oc_ value of the chemical substances (Nishioka et al. 2018) would be recommended for water bodies in Japan even if the sample size were to increase.

There are no clear guidelines for setting the PEC of a chemical substance for spatially extensive areas. As such, high concentrations that can cover the majority of public water bodies are generally used for PEC (Ministry of the Environment, Japan 2014). When determining the PEC from measured values, the maximum value is often used if the number of data is limited. If enough data are available, high percentile values (e.g., 95th percentile) are often used (Miura et al. 2005). Similarly, when calculating the PEC using environmental exposure models, high percentile values are often used (Jensen et al. 2007; Capdevielle et al. 2008); thus, the quantile estimation accuracy of environmental exposure models is essential for chemical risk assessment regardless of the model type. In this study, we used more than 10 000 samples and showed that the 95th and 90th percentiles of the measured values can be predicted with high accuracy by avoiding extreme values. Therefore, we propose that the strategy of using the 95th and 90th percentiles from AIST‐SHANEL to derive the PEC allows for a practical environmental risk assessment covering a wide range of environments.

Conversely, for individual sites, even when conducting analyses of the annual site mean data of rivers, which have the best analytical accuracy, 18% of the data points fall outside the 10× factor range; this could cause issues with regard to high accuracy estimations of chemical concentrations, depending on the site. Consequently, we believe that there is a need to organize the characteristics of regional information for sites with a large separation between estimated and measured values, and to further improve the model. For instance, the model may be improved by using big data analytics for the measured values of chemicals other than LAS to create an exposure assessment model that can be applied to various physical properties of the chemicals.

In conclusion, we evaluated the accuracy of AIST‐SHANEL in major rivers throughout Japan using 3 years of environmental monitoring data to develop a strategy for the effective application of AIST‐SHANEL to environmental risk assessments. Our results support the use of AIST‐SHANEL as an effective tool for estimating chemical concentrations. Thus, the model can be practically applied toward high accuracy environmental risk assessments using the 95th percentile of estimated values as the PEC. Moreover, the estimation ability was particularly good when annual mean river data were used. The optimal traction coefficient of LAS was 100 m^‒1^ or higher, similar to that of a previous report (Nishioka et al. 2018). In recent years, worldwide developments in chemical management have been reported following the founding of the SAICM; thus, there is a demand to understand the spatiotemporal exposure distribution conditions of many different chemicals at a national scale. As such, the results of the present research hold promise for practical assessments of such chemicals.

## Data Accessibility

Data used in this study are available in the Supplemental Data. The measured concentration of LAS in major Japanese rivers can be accessed on the website of the Ministry of the Environment, Japan under “Public water area ‐ Water quality measurement result” (in Japanese), http://www.env.go.jp/water/suiiki/

## SUPPLEMENTAL DATA

Supplemental data (1 file) contains tables of the raw data of estimated and measured concentrations of LAS.

## Supporting information

This article contains online‐only Supplemental Data.
